# Comparison of peripheral and cerebral vascular function between premenopausal, early and late postmenopausal females

**DOI:** 10.1113/EP090813

**Published:** 2023-01-09

**Authors:** Stefanie L. Ruediger, Faith K. Pizzey, Jodie L. Koep, Jeff S. Coombes, Christopher D. Askew, Tom G. Bailey

**Affiliations:** ^1^ Physiology and Ultrasound Laboratory in Science and Exercise Centre for Research on Exercise, Physical Activity and Health School of Human Movement and Nutrition Sciences The University of Queensland Brisbane Queensland Australia; ^2^ Children's Health and Exercise Research Centre, Sport and Health Sciences College of Life and Environmental Sciences University of Exeter Exeter UK; ^3^ VasoActive Research Group School of Health University of the Sunshine Coast Sippy Downs Queensland Australia; ^4^ Sunshine Coast Health Institute Sunshine Coast Hospital and Health Service Birtinya Queensland Australia; ^5^ School of Nursing Midwifery and Social Work The University of Queensland Brisbane Queensland Australia

**Keywords:** arterial stiffness, cerebrovascular reactivity, internal carotid artery, menopause, oestrogen, vascular function

## Abstract

The risk of cardiovascular and cerebrovascular disease increases in ageing females, coinciding with the onset of menopause. Differences in peripheral and cerebrovascular function across menopausal stages, however, are poorly characterized. The aim of this study was to compare peripheral and cerebrovascular function between healthy premenopausal (PRE), early (1–6 years after final menstrual period; E‐POST) and late (>6 years after final menstrual period; L‐POST) postmenopausal females. We also explored the association between reproductive hormones, NO bioavailability and cerebrovascular function. In 39 females (40–65 years of age), we measured arterial stiffness, brachial artery flow‐mediated dilatation, and cerebrovascular reactivity (CVR) to hypercapnia in the middle (MCAv) and internal (ICA) carotid arteries. Follicle‐stimulating hormone, estradiol, progesterone and plasma nitrate and nitrite concentrations were also measured. Years since final menstrual period (PRE, 0 ± 0 years; E‐POST, 3 ± 1 years; L‐POST, 11 ± 4 years; *P* < 0.001) and estradiol levels (PRE, 145.5 ± 65.6 pg ml^−1^; E‐POSTm 30.2 ± 81.2 pg ml^−1^; L‐POST, 7.7 ± 11.3 pg ml^−1^; *P* < 0.001) were different between groups. All groups exceeded the guidelines for recommended physical activity. There were no group differences in blood pressure (*P* = 0.382), arterial stiffness (*P* = 0.129), flow‐mediated dilatation (*P* = 0.696) or MCAv CVR (*P* = 0.442). The ICA CVR blood flow response was lower in PRE compared with L‐POST (26.5 ± 19.2 vs. 47.8 ± 12.6%; *P* = 0.010), but after adjusting for age these differences were no longer present. Flow‐mediated dilatation (*r* = 0.313, *P* = 0.105) and ICA CVR (*r* = −0.154, *P* = 0.495) were not associated with the estradiol concentration. There were no associations between the estradiol concentration and NO bioavailability. These results suggest that in healthy, physically active early and late postmenopausal females, vascular and cerebrovascular function is generally well preserved.

## Abstract (highlights)

- **What is the central question of this study?**
  We sought to investigate whether peripheral and cerebrovascular function are impaired in early and late postmenopausal females compared with premenopausal females, while also accounting for nitric oxide and estradiol levels.
- **What is the main finding and its importance?**
  We observed no differences in peripheral vascular and cerebrovascular function between healthy and physically active premenopausal females and early and late postmenopausal females. Our findings contradict previous cross‐sectional observations of vascular and cerebrovascular dysfunction across menopause. Longitudinal studies assessing vascular and cerebrovascular outcomes across the menopausal transition are warranted.

## 1 INTRODUCTION

Menopause is associated with increased risk of cardiovascular and cerebrovascular diseases (De Kat et al., 2017; Haast et al., 2012). Based on epidemiological data, early onset menopause and longer duration of menopause have also been shown to increase the risk of fatal and non‐fatal cardiovascular disease, including stroke, and earlier overall mortality in postmenopausal females (Muka et al., 2016; Zhu et al., 2019). Rapid declines in circulating oestrogen during the menopause are suggested to have negative impacts on vascular structure and function, including in the peripheral and cerebral circulation (Duckles & Krause, 2007; Moreau et al., 2012). Oestrogen acts directly on the endothelium and can influence the production of vasoactive molecules, including NO (Chambliss & Shaul, 2002; Gavin et al., 2009), which plays an important role in vascular endothelial function (Green et al., 2014; Vallance & Chan, 2001). A rapid decline in oestrogen levels could negatively impact vascular function and stiffness in postmenopausal females and accelerate vascular ageing. Understanding the impact of menopause on vascular ageing is important because its determinants, including endothelial dysfunction and arterial stiffening, both independently predict the risk of future cardiovascular disease (CVD) events and mortality (D'Agostino et al., 2008; Kim et al., 2022; Thijssen et al., 2016).

A central change during vascular ageing is arterial stiffening, leading to alterations in central haemodynamics, increased pulse pressure and endothelial dysfunction, which, in turn, contributes to arterial stiffening (Lyle & Raaz, 2017; Shirwany & Zou, 2010; Thijssen et al., 2016). Cross‐sectional studies show that the transition from pre‐ to postmenopause is associated with endothelial dysfunction and increased arterial stiffness (Moreau et al., 2012; Taddei et al., 1996; Zaydun et al., 2006). The phase of menopause might also be important, including whether females are in early [1–6 years since final menstrual period (FMP)] or late (≥6 years since FMP) postmenopause (Harlow et al., 2012). For example, arterial stiffness, as measured by pulse wave velocity (PWV), is increased in post‐ compared with premenopausal females (Takahashi et al., 2005; Zaydun et al., 2006). However, PWV was not different between early postmenopausal and premenopausal females (Takahashi et al., 2005). Menopause might augment the increase in arterial stiffness in females, but given that natural ageing also leads to arterial stiffening and that vascular changes develop over time, it is difficult to separate the effect of menopause, including decreases in oestrogen, and age, on arterial stiffening (Takahashi et al., 2005; Zaydun et al., 2006). Direct measures of hormone concentration are needed to help to distinguish between the effects of menopause and ageing.

Endothelial function, measured via brachial artery flow‐mediated dilatation (FMD) has been shown to decline throughout the stages of menopause in some (Brislane et al., 2020; Holder et al., 2019; Moreau et al., 2012), but not all (Debray et al., 2022) previous studies. Comparisons between studies are difficult owing to methodological differences in accounting for menstrual cycle phase, characterizing menopause, including adherence to menopausal criteria (STRAW+10) (Harlow et al., 2012), and the variable and often large age differences between menopausal groups. The impact of menopausal stage on arterial stiffness and endothelial function, while accounting for changes in reproductive hormone levels and NO bioavailability, warrants further investigation.

The effect of menopause on cerebrovascular health has received little attention. In a systematic review, we highlighted that the effect of menopausal status on middle cerebral artery velocity (MCAv; both at rest and reactivity to hypercapnia), is inconclusive (Ruediger et al., 2021). Inconsistent menopausal and menstrual stage definition and lack of adherence to current methodological guidelines for cerebrovascular assessment (Thomas et al., 2015; Willie et al., 2011) might have contributed to the inconsistent outcomes. When studies are standardized and conducted in line with STRAW+10 criteria, findings suggest that menopause might adversely impact extracranial, but not intracranial cerebrovascular health. For example, Brislane et al. (2020) report negligible differences in MCAv cerebrovascular reactivity (CVR) to hypercapnia, whereas internal carotid artery (ICA) CVR is reduced in postmenopausal compared with premenopausal females (Iwamoto et al., 2020). This indicates that alterations in cerebral blood flow during menopause might differ between the extra‐ and intracranial cerebrovasculature. To date, no study has investigated intracranial and extracranial cerebrovascular function comprehensively in both pre‐ and postmenopausal females.

The primary aim of this study was to compare peripheral and cerebral vascular function between premenopausal, early and late postmenopausal females. When accounting for the STRAW+10 criteria (Harlow et al., 2012), we hypothesized that late postmenopausal females would have lower peripheral vascular and cerebrovascular function compared with premenopausal and early postmenopausal females. We also aimed to assess the associations between reproductive hormone status and (1) measures of peripheral and cerebrovascular function and (2) NO bioavailability in all pre‐ and postmenopausal females.

## 2 METHODS

### 2.1 Ethical approval

This study was approved by the University of Queensland Research Ethics Committee (approval no. 2018002256) in accordance with the *Declaration of Helsinki*, except for registration in a database. Before enrolling in the study, written informed consent was provided by all participants.

### 2.2 Participants

Thirty‐nine healthy females aged between 40 and 65 years were recruited from the greater Brisbane area. The participants were all non‐smokers, free of any known cerebrovascular, cardiovascular or any other chronic disease. Participants were excluded if their body mass index was >35 kg/m^2^ or if they were taking any hormone replacement therapy other than the oral contraceptive pill or hormonal intra‐uterine devices in the premenopausal females. All premenopausal females experienced a regular menstrual cycle, occurring from 26 to 35 days in length during the last 3 months before the study, and were tested during the luteal phase of their cycle (Harlow et al., 2013; Mihm et al., 2011). The luteal phase was selected to attain a higher oestrogen concentration in the premenopausal females. Based on self‐report, female participants were classified as premenopausal (PRE), early postmenopausal (E‐POST; 1–6 years since FMP) or late postmenopausal (L‐POST, >6 years post FMP) using the STRAW+10 criteria (Harlow et al., 2012). Perimenopausal females were not included in this study. Postmenopause was confirmed by hormone analysis. Physical activity levels (in metabolic equivalent (MET) minutes per week) were determined using the Active Australia Survey (AIHW, 2003). Walking was defined as 3.5 METs, moderate activities as four METs and vigorous activities as seven METs (AIHW, 2003).

### 2.3 Experimental procedures

All participants attended testing after fasting for ≥3 h and were asked to refrain from any alcohol, caffeine and nitrate‐rich foods for 12 h before testing and to not engage in any vigorous exercise for 12 h before their visit. After initial screening and questionnaires, venous blood samples were obtained. After 15 min of supine rest in a temperature‐controlled room (∼23°C), resting blood pressure (BP), arterial stiffness and brachial FMD were measured. This was followed by simultaneous assessments of intra‐ and extracranial cerebral blood flow reactivity. All vascular and cerebrovascular assessments were performed with the participant remaining in a supine position.

### 2.4 Arterial stiffness and blood pressure

#### 2.4.1 Pulse wave analysis

Brachial artery blood pressure was measured in triplicate, using an automated device (SphygmoCor XCEL; AtCor Medical, West Ryde, NSW, Australia). After BP measurement, the arm cuff was inflated to slightly above diastolic pressure for 10 s. A corresponding aortic pressure wave form was generated, from which the augmentation index (AIx), central systolic (cSBP), diastolic (cDBP) and central pulse (cPP) pressures were derived. The AIx was corrected for a heart rate at 75 beats min^−1^ (AIx75), because AIx is affected by heart rate (Wilkinson et al., 2000).

#### 2.4.2 Pulse wave velocity

To assess PWV, pulse waves were monitored at the carotid artery by a hand‐held tonometer, and simultaneously, pulse waves were monitored at the femoral artery using a low‐pressure pneumatic thigh cuff, which remained partly inflated over a period of 20–60 s. The distance between (1) the top of the cuff to the inguinal ligament; (2) the top of the cuff to the sternal notch; and (3) the carotid pulse to the sternal notch were measured individually for each participant. The femoral and carotid waveforms were assessed, and the system automatically determined the pulse transfer time (in metres per second) from the femoral to the carotid artery by calculating the ratio of the distance between the carotid and femoral arteries to the transit time. Measurements were based on 10 s pulse wave traces that were free of artefact and met the quality control threshold of the SphygmoCor Xcel device for pulse‐to‐pulse variability. Measures were performed in duplicate, with a third measurement if PWV differed by >0.5 m s^−1^.

### 2.5 Peripheral vascular function

#### 2.5.1 Brachial artery FMD

Flow‐mediated dilatation was examined in response to 5 min of ischaemia, following gold‐standard assessment and analysis guidelines (Thijssen et al., 2019). Participants were assessed in the supine position, using a rapid occlusion cuff (Rapid cuff inflation system E20; Hokanson, Bellevue, WA, USA), placed distal to the imaged artery on the right forearm of the participant. The cuff pressure was standardized to 220 mmHg. Baseline diameter, blood flow and velocity of the brachial artery were collected for 1 min before cuff occlusion, via 12 MHz Doppler ultrasound (Terason Usmart 3300; Teratech Corporation, Burlington, MA, USA). The cuff was then inflated for 5 min before rapid deflation, Brachial artery diameter, blood flow velocity and shear rate were recorded for 3 min after cuff deflation.

### 2.6 Cerebrovascular blood flow

#### 2.6.1 Cerebrovascular reactivity

The CVR protocol consisted of a 2 min baseline, breathing room air, followed by 5 min of hypercapnia. During hypercapnia, 5% CO_2_ was administered with 21% O_2_ and balanced nitrogen. A three‐way valve (Hans Rudolph, Shawnee, KS, USA) was used to switch from ambient air to the 5% CO_2_ mixture using a 170 L Douglas bag (Hans Rudolph). Participants were instructed to breathe normally during hypercapnia and the baseline periods. End‐tidal carbon dioxide ($P_{\mathrm{ETC}O_{2}}$) and blood pressure were measured continuously throughout. At rest and during CVR, both intracranial and extracranial assessments were undertaken and are described below.

#### 2.6.2 Intracranial arteries

Cerebral blood flow was assessed from measures of the right middle (MCAv) and left posterior (PCAv) cerebral artery flow velocities using transcranial Doppler ultrasound (ST 3; Spencer Technologies, Redmond, WA, USA). A 2 MHz Doppler probe was placed on each side of the temporal window, secured, and adjusted until an optimal signal was identified. The probes were held in place using a headband strap to prevent movement of the Doppler probe and maintain insonation angle accuracy.

#### 2.6.3 Extracranial arteries

The right ICA diameter and mean blood velocity were measured using a 12 MHz Doppler probe and a high‐resolution Duplex ultrasound (Terason Usmart 3300; Teratech, Burlington, MA, USA). The artery was identified and assessed following the recent guidelines (Thomas et al., 2015). The arterial diameter was measured with B‐mode imaging, and an insonation angle of 60° was maintained throughout each test. After optimization of the image, images of the artery and the associated velocity waveforms were recorded simultaneously during baseline and the hypercapnic protocol.

### 2.7 End‐tidal carbon dioxide and blood pressure

All cerebrovascular measurements were assessed along with beat‐by‐beat blood pressure, heart rate (Finapres Nova; Finapres Medical Systems, Enschede, Netherlands) and $P_{\mathrm{ETC}O_{2}}$ (PowerLab 8 Gas Analyzer; ADInstruments, Colorado Springs, CO, USA). All data were recorded using LabChart 8 (ADInstruments) and stored offline for later analysis.

### 2.8 Blood analysis

#### 2.8.1 Nitric oxide

Given that NO is a radical gas with a short half‐life, measurements of the stable NO metabolites, nitrate (NO_3_^−^) and nitrite (NO_2_^−^) were used to estimate NO production and bioavailability. A NO analyser purge vessel system (Sievers 280i; Zysense, Weddington, NC, USA) was used for the plasma analysis. Nitrite was analysed according to methods developed by Nagababu & Rifkind (2010), using chemiluminescence. The nitrate and nitrite concentrations were derived from the integral of the NO‐generated signal over time compared with those obtained for standard concentrations of nitrate and nitrite via the NO analyser computer software (Zysense). Concentrations were calculated using the area under the curve provided by the software and the following:

$$\begin{array}{ccc} & & \mathrm{Nitrite}\;\mathrm{or}\;\mathrm{nitrate}\;\mathrm{level}=\frac{\mathrm{Area}-y-\mathrm{intercept}}{\mathrm{Slope}}\\ & & \;\times \;\frac{\mathrm{Volumes}\;\mathrm{of}\;\mathrm{standard}\;\mathrm{injected}\;(\mathrm{in}\;\mathrm{millilitres})}{\mathrm{Volume}\;\mathrm{of}\;\mathrm{plasma}\;\mathrm{injected}\;(\mathrm{in}\;\mathrm{millilitres})}\left(\times \mathrm{Dilution}\;\mathrm{factor}\right) \end{array}$$

For nitrate only, the dilution factor was four.

#### 2.8.2 Reproductive hormones

Plasma concentrations of follicle‐stimulating hormone (FSH), progesterone and estradiol were analysed using s Cobas e 411 analyser and respective analysis kits (Elecsys FSH, Elecsys Progesterone and Elecsys Estradiol III; Cobas; Roche Diagnostics, Mannheim, Germany). The measurement range was as follows: estradiol, 18.4–11,010 pmol L^−1^ (5–3,000 pg ml^−1^); progesterone, 0.159–191 nmol L^−1^ or 0.05–60 ng ml^−1^; and FSH, 0.100–200 mIU ml^−1^. All samples were analysed in duplicate. A third sample was run if there was a difference of >0.5% in the concentration in the two samples.

### 2.9 Data processing and analysis

All Doppler ultrasound recordings (brachial artery and ICA) were analysed using a customized edge‐detection and wall‐tracking software package (Bloodflow Analysis 5.1; Woodman et al., 2001). From recordings of the synchronized arterial diameter and blood velocity data, blood flow was calculated at 30 Hz. Shear rate was calculated as four times the mean blood velocity/vessel diameter. Brachial FMD was calculated as [(peak diameter minus baseline diameter)/baseline diameter] and expressed as a percentage change in vessel diameter. The percentage FMD was also corrected for variability in baseline diameter between groups and expressed as the scaled FMD (Atkinson & Batterham, 2013).

#### 2.9.1 Intracranial blood flow

The MCAv, PCAv, blood pressure and $P_{\mathrm{ETC}O_{2}}$ were exported from LabChart as 1 s‐averaged bins to Excel (Microsoft 365, Redmond, WA, USA). Baseline values were averaged over 1 min of supine rest. The cerebrovascular response was calculated as both absolute and relative (percentage) change from baseline in MCAv, per unit increase (in millimetres of mercury) in $P_{\mathrm{ETC}O_{2}}$. The peak was determined as the peak response, wherever it occurred rather than a set time point, in line with recent findings and recommendations from our laboraory (Koep et al., 2022). The cerebrovascular conductance index (CVCi) of the MCA was calculated as MCAv/mean arterial pressure, cerebrovascular resistance index (CVRi) as mean arterial pressure/MCAv, and pulsatility index (PI) as (maximum MCAv minus minimum MCAvmin)/mean MCAv.

#### 2.9.2 Extracranial blood flow

The ICA blood flow and shear rate were calculated automatically via the analysis software (Bloodflow Analysis 5.1; Woodman et al., 2001), using ICA diameter and blood velocity data collected at rest and during CVR. All data were imported into General Purpose Data Graphing Software (v.1). The approach was based on the work of previous groups (Carr et al., 2020; Carter et al., 2016; Hoiland et al., 2017). The software automatically smoothed the data and calculated baseline values and the peak response during CVR. Automatically detected thresholds and peaks were assessed visually to ensure that they met the following criteria: (1) the algorithm‐detected threshold point occurred before the peak value; and (2) the variable did not decrease below the algorithm‐detected threshold point before the peak value occurring (Carr et al., 2020; Carter et al., 2016). Shear rate area under the curve (SRAUC) was calculated from the onset of hypercapnia to peak diameter using GraphPad Prism 8 (GraphPad Software, San Diego, CA, USA).

### 2.10 Statistical analysis

All data were analysed using Excel (Microsoft 365) and SPSS Statistics 27 (IBM SPSS, Armonk, NY, USA). GraphPad Prism 8 (GraphPad Software) was used to visualize data. Normality and homogeneity of variance were checked with the Shapiro–Wilk test and Levene's test. In normally distributed data, a one‐way ANOVA was used to assess differences in all peripheral vascular, cerebrovascular and blood outcomes between groups (PRE, E‐POST and L‐POST). To investigate the effect of age and estradiol on the outcomes, analysis of covariance (ANCOVA) was used, with age and estradiol as covariates independently in two separate models and the respective outcomes as fixed factors. To investigate the effect of physical activity on the outcomes, ANCOVA was also used, with physical activity as a covariate and brachial FMD and ICA CVR as fixed factors. If applicable, Tukey's post‐hoc analysis was used to identify differences in outcomes between the group means. The Pearson correlation coefficient was used to investigate associations between CVR, blood hormone levels and NO concentration, and between age and CVR and age and brachial FMD. For further analysis, hormone data were logarithmically transformed, owing to the skewness of the data. All data are reported as the mean ± SD unless otherwise stated. Statistical significance was accepted at *P* ≤ 0.05.

## 3 RESULTS

### 3.1 Participant characteristics and peripheral vascular function

Table 1 presents the characteristics of the participants. Reproductive hormone levels confirmed postmenopause and were lower in postmenopausal compared with premenopausal groups (*P* < 0.001). Sixty per cent of premenopausal females were not using any hormonal contraceptives, 30% were on oral contraceptives, and one person (10%) had an intra‐uterine device. All but three participants (one in each group) met the recommended Australian physical activity guidelines and were classified as sufficiently physically active (AIHW, 2003). Physical activity was high in all groups, with L‐POST displaying the highest level of MET minutes per week. However, there was no influence of physical activity on brachial FMD or ICA CVR.

**TABLE 1:** Baseline characteristics for premenopausal (PRE), early (E‐POST) and late (L‐POST) postmenopausal females.

**Characteristics**	**PRE (*n* = 10)**	**E‐POST (*n* = 15)**	**L‐POST (*n* = 14)**	**ANOVA *P*‐value**
Age, years	47 ± 3^†^	55 ± 4^‡^	60 ± 3	<0.001
Years since FMP	0 ± 0* ^†^	3 ± 1^‡^	11 ± 4	<0.001
Height, cm	171 ± 9	169 ± 5	167 ± 5	0.278
Body mass, kg	73 ± 13	72 ± 15	68 ± 11	0.694
BMI, kg m^−2^	24.6 ± 2.6	25 ± 5.1	24.5 ± 3.4	0.948
Estradiol, pg ml^−1^	154.1 (117.8–194.1)* ^†^	6.6 (5.1–8.1)	0 (0–8.0)	<0.001
FSH, mIU ml^−1^	5.8 (2.6 to 29.5)^†^	80.9 (61.2 to 103.2)	87.9 (73.2–104.1)	<0.001
Progesterone, μg ml^−1^	13.1 (0.5–18.4)* ^†^	0.09 (0.07–0.1)	0.08 (0.06–0.1)	<0.001
Physical activity, MET min week^−1^	1,621 ± 1,035	1,590 ± 1,168	2,573 ± 1,586	0.098

*Note*: Hormones are reported as the median and interquartile range.

Abbreviations: BMI, body mass index; FMP, final menstrual period; FSH, follicle‐stimulating hormone; MET, metabolic equivalent.

* Significant difference (*P* < 0.05) between PRE and E‐POST.

^†^ Significant difference (*P* < 0.05) between PRE and L‐POST.

^‡^ Significant difference (*P* < 0.05) between E‐POST and L‐POST.

Brachial FMD was excluded for one E‐POST participant owing to technical error. There were no differences between groups for blood pressure, arterial stiffness or brachial FMD (Table 2). Brachial FMD was not correlated with age (*r* = −0.196, *P* = 0.238).

**TABLE 2:** Blood pressure, arterial stiffness and brachial artery flow‐mediated dilatation in premenopausal (PRE), early (E‐POST) and late (L‐POST) postmenopausal females.

Parameter	PRE	E‐POST	L‐POST	ANOVA *P*‐value
**Blood pressure and heart rate**				
**Resting SBP, mmHg**	114 ± 12	119 ± 14	118 ± 9	0.505
**Resting DBP, mmHg**	67 ± 83	72 ± 8	70 ± 8	0.371
**Resting MAP, mmHg**	82 ± 9	88 ± 10	86 ± 7	0.382
**HR, beats min^−1^**	56 ± 6	57 ± 8	56 ± 9	0.967
**Central SBP, mmHg**	102 ± 12	109 ± 14	108 ± 8	0.258
**Central DBP, mmHg**	68 ± 8	72 ± 9	71 ± 7	0.364
**Central PP, mmHg**	34 ± 6	38 ± 9	37 ± 6	0.531
**Arterial stiffness**				
**PWV, cm s^−1^**	6.4 ± 2.7	6.8 ± 2.8	8.6 ± 3.0	0.129
**AIx, a.u**.	20.6 ± 14.7	27.4 ± 11.3	27.6 ± 9.0	0.227
**AIx75**	11.5 ± 16.6	18.7 ± 12.4	16.6 ± 11.3	0.350
**Pulse transit time, s**	116.4 ± 65.6	96.7 ± 60.6	76.5 ± 55.4	0.286
**Brachial FMD**				
***D*_base_, cm**	0.35 ± 0.03	0.34 ± 0.05	0.36 ± 0.07	0.823
***D*_peak_, cm**	0.38 ± 0.03	0.36 ± 0.05	0.37 ± 0.05	0.770
**Change, cm**	0.02 ± 0.01	0.02 ± 0.01	0.02 ± 0.01	0.470
**Percentage FMD**	7.0 ± 3.7	6.1 ± 3.5	5.9 ± 3.2	0.696
**Time to peak, s**	80.0 ± 53.6	82.8 ± 59.6	55.6 ± 46.2	0.304
**SRAUC, s^−1^ ×10^3^**	20.2 ± 68.7	20.7 ± 12.4	15.9 ± 11.6	0.463

Abbreviations: AIx, central augmentation index; *D*_base_, brachial artery baseline diameter; *D*_peak_, brachial artery peak diameter; DBP, diastolic blood pressure; HR, heart rate; MAP, mean arterial pressure; PP, pulse pressure; PWV, pulse wave velocity; SBP, systolic blood pressure; SRAUC, shear rate area under the curve.

### 3.2 Cerebrovascular reactivity

#### 3.2.1 Intracranial CVR

Data for intracranial analysis are included for 35 females (PRE, 8; E‐POST, 12; L‐POST, 15), owing to the inability to obtain a sufficient MCAv signal in four participants. Table 3 shows that MCAv increased by 33–40% from baseline to peak during hypercapnia in all three groups (*P* < 0.001). Changes in the MCA blood flow in response to hypercapnia were not different between groups. There were no differences between groups for any other parameters. When age and estradiol were used as covariates independently, the results did not change.

**TABLE 3:** Intracranial response to hypercapnia in premenopausal (PRE), early (E‐POST) and late (L‐POST) postmenopausal females.

Parameter	Measure (units)	PRE	E‐POST	L‐POST	ANOVA *P*‐value
**MCAv**	Baseline (cm s^−1^)	76.9 ± 12.9	71.4 ± 20.2	73.1 ± 12.7	0.745
	Peak (cm s^−1^)	102.3 ± 12.0	95.1 ± 20.4	101.4 ± 13.7	0.520
	Change (cm s^−1^)	25.4 ± 3.9	27.9 ± 14.4	28.3 ± 8.5	0.831
	%Change	34.0 ± 8.9	37.0 ± 20.4	40.1 ± 14.1	0.726
**MCAv CVRi**	Baseline (mmHg cm^−1^ s)	0.8 ± 0.2	0.7 ± 0.2	0.7 ± 0.2	0.807
	Peak (mmHg cm^−1^ s)	1.0 ± 0.2	0.9 ± 0.2	0.9 ± 0.2	0.835
	Change (mmHg cm^−1^ s)	0.2 ± 0.2	0.2 ± 0.2	0.1 ± 0.2	0.915
	%Change	26.7 ± 5.7	29.9 ± 10.6	28.0 ± 12.3	0.775
**MCAv CVCi**	Baseline (cm s^−1^ mmHg^−1^)	1.3 ± 0.2	1.5 ± 0.3	1.4 ± 0.3	0.716
	Peak (cm s^−1^ mmHg^−1^)	1.6 ± 0.3	1.6 ± 0.4	1.5 ± 0.3	0.779
	Change (cm s^−1^ mmHg^−1^)	0.2 ± 0.1	0.2 ± 0.1	0.2 ± 0.1	0.349
	%Change	14.9 ± 12.9	10.9 ± 11.0	10.4 ± 11.4	0.689
**MCAv PI**	Baseline	0.7 ± 0.1	0.9 ± 0.4	0.8 ± 0.1	0.649
	Peak	1.3 ± 1.1	1.1 ± 0.7	0.9 ± 0.1	0.580
	Change	0.5 ± 1.1	0.2 ± 0.3	0.1 ± 0.1	0.349
	%Change	20.2 ± 9.7	24.1 ± 13.6	15.3 ± 8.3	0.128
**MCAv CVR**	Relative (% mmHg^−1^)	3.0 ± 0.9	4.1 ± 3.0	3.4 ± 1.2	0.442
	Absolute (cm s^−1^ mmHg^−1^)	2.2 ± 0.4	2.8 ± 1.9	2.4 ± 0.7	0.595
**PCAv**	Baseline (cm s^−1^)	49.3 ± 18.0	43.7 ± 10.8	45.1 ± 11.5	0.626
	Peak (cm s^−1^)	66.8 ± 18.7	55.8 ± 11.4	60.7 ± 15.3	0.256
	Change (cm s^−1^)	17.5 ± 3.8	12.7 ± 5.6	17.0 ± 6.7	0.090
	%Change	27.0 ± 8.5	26.9 ± 9.1	27.8 ± 8.0	0.959
**MAP**	Baseline (mmHg)	96 ± 9	103 ± 13	106 ± 16	0.195
	Peak (mmHg)	113 ± 13	118 ± 16	126 ± 138	0.097
	Change (mmHg)	15 ± 9	15 ± 10	19 ± 7	0.551
	%Change	17.4 ± 6.4	15.4 ± 9.6	20.5 ± 13.5	0.454
$P_{\mathrm{ETC}O_{2}}$	Baseline (mmHg)	40.1 ± 4.2	43.0 ± 4.6	41.8 ± 5.0	0.330
	Peak (mmHg)	51.6 ± 3.4	54.4 ± 5.5	53.7 ± 4.3	0.386
	Change (mmHg)	11.6 ± 1.2	11.4 ± 3.6	11.5 ± 2.6	0.985
	%Change	27.0 ± 8.5	26.9 ± 9.1	27.8 ± 8.0	0.442

Abbreviations: CVCi, cerebrovascular conductance index; CVR, cerebrovascular reactivity; CVRi, cerebrovascular resistance index; MAP, mean arterial pressure; MCAv, middle cerebral artery velocity; PCAv, posterior cerebral artery; $P_{\mathrm{ETC}O_{2}}$, end‐tidal partial pressure of carbon dioxide; PI, pulsatility index.

#### 3.2.2 Extracranial CVR

Data for extracranial analysis are included for 34 females (PRE, 9; E‐POST, 12; L‐POST, 13), owing to the inability to obtain a satisfactory ultrasound image of the ICA in five participants. The ICA velocity, diameter, blood flow and shear rate increased from baseline during hypercapnia (Table 4). The absolute change in ICA blood flow was lower in PRE compared with E‐POST (*P* = 0.035) and L‐POST (*P* = 0.016). The relative change in ICA blood flow was lower in PRE when compared with L‐POST (*P* = 0.010), with no differences in ICA diameter, velocity or shear rate between groups. When using age and estradiol as covariates independently, there was no difference in relative or absolute change of ICA blood flow between groups. The ICA CVR was not correlated with age (*r* = 0.264, *P* = 0.145), and age or estradiol did not influence any of the other outcomes.

**TABLE 4:** Extracranial cerebrovascular response to hypercapnia in premenopausal (PRE), early (E‐POST) and late (L‐POST) postmenopausal females.

Parameter	Measure (units)	PRE	E‐POST	L‐POST	ANOVA *P*‐value
**ICA diameter**	Baseline (cm)	0.5 ± 0.1	0.6 ± 0.1	0.7 ± 0.2	0.080
	Peak (cm)	0.6 ± 0.1	0.7 ± 0.1	0.7 ± 0.2	0.067
	Change (cm)	0.02 ± 0.01	0.07 ± 0.18	0.02 ± 0.01	0.447
	%Change	3.4 ± 1.3	3.5 ± 1.5	4.5 ± 1.9	0.262
**ICA velocity**	Baseline (cm s^−1^)	39.5 ± 12.9	38.4 ± 10.0	36.0 ± 9.9	0.772
	Peak (cm s^−1^)	54.5 ± 17.7	53.6 ± 13.4	50.1 ± 13.6	0.769
	Change (cm s^−1^)	10.0 ± 6.4	14.9 ± 5.6	17.3 ± 5.5	0.062
	%Change	37.9 ± 17.6	40.6 ± 14.9	40.4 ± 17.3	0.940
**ICA blood flow**	Baseline (ml s^−1^)	10.1 ± 5.7	13.9 ± 4.9	11.0 ± 4.5	0.241
	Peak (ml s^−1^)	12.4 ± 6.2	18.7 ± 7.0	15.8 ± 5.5	0.131
	Change (ml s^−1^)	2.3 ± 1.5* ^†^	4.8 ± 2.4	4.9 ± 1.6	0.013
	%Change	26.5 ± 19.2^†^	33.5 ± 12.6	47.8 ± 12.6	0.010
**ICA Shear rate**	Baseline (s^−1^)	185.2 ± 91.5	278.9 ± 103.4	257.5 ± 123.2	0.233
	Peak (s^−1^)	235.4 ± 149.9	389.13 ± 160.5	358.8 ± 168.5	0.198
	Change (s^−1^)	60.5 ± 61.6	110.2 ± 68.4	110.0 ± 52.5	0.241
	%Change (s^−1^)	29.7 ± 16.3	39.2 ± 17.7	48.3 ± 14.1	0.100
**SRAUC, s^−1^ × 10^3^**		104.2 ± 39.7	88.6 ± 36.9	70.7 ± 33.8	0.236

Abbreviations: ICA, internal carotid artery; SRAUC, shear rate area under the curve.

* Significant difference (*P* < 0.05) between PRE and E‐POST.

^†^ Significant difference (*P* < 0.05) between PRE and L‐POST.

### 3.3 Nitric oxide and hormones

**FIGURE 1:**
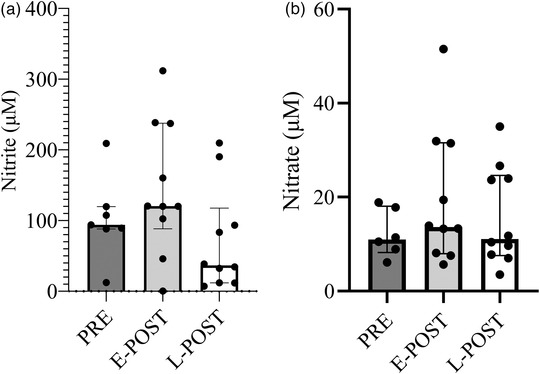
(a,b) Median and interquartile range for (a) nitrite and (b) nitrate concentrations in premenopausal (PRE), early (E‐POST) and late (L‐POST) postmenopausal females. Nitrite (μM): PRE, 102.8 ± 58.1; E‐POST, 145.7 ± 94.1; L‐POST, 71.3 ± 73.8; *P* = 0.128. Nitrate (μM): PRE, 12.3 ± 5.0; E‐POST, 19.6 ± 14.5; L‐POST, 15.9 ± 10.5; *P* = 0.465.

Plasma nitrate and nitrite concentrations were not different between groups (Figure 1). As mentioned previously, there was a difference between groups for estradiol, progesterone and FSH (Table 1). There were no associations between estradiol and plasma NO (nitrate, *r* = −0.156, *P* = 0.468; nitrite, *r* = −0.118, *P* = 0.468) or progesterone and plasma NO (nitrate, *r* = −0.156, *P* = 0.468; nitrite, *r* = −0.164, *P* = 0.433) or FSH and plasma NO (nitrate, *r* = 0.276, *P* = 0.191; nitrite, *r* = 0.137, *P* = 0.512). There were also no associations between estradiol and brachial FMD (*r* = −0.106, *P* = 0.590) or ICA CVR (*r* = −0.085, *P* = 0.708), nor between progesterone and brachial FMD (*r* = −0.118, *P* = 0.551) or ICA CVR (*r* = −0.059, *P* = 0.794), or FSH and brachial FMD (*r* = −0.0182, *P* = 0.353) or ICA CVR (*r* = −0.012, *P* = 0.957). Owing to the skewness of the hormone data (scatterplots are provided in the Supplementary Material) and concerns that use of linear statistics might limit interpretation, logarithmic transformation was used, and correlations were assessed in the postmenopausal women separately. There were still no associations between brachial FMD and estradiol or progesterone. Follicle‐stimulating hormone was correlated with brachial FMD in the postmenopausal females when using the logarithmically transformed data (*r* = −0.592, *P* = 0.006). Estradiol was correlated with ICA CVR when looking at the postmenopausal group separately (*r* = 0.667, *P* = 0.035). The other hormones were not correlated with ICA CVR.

## 4 DISCUSSION

This is the first study to compare peripheral, intra‐ and extracranial vascular function comprehensively between premenopausal, early postmenopausal and late postmenopausal females in line with STRAW+10 criteria, including NO and reproductive hormone analysis. There were no differences in resting blood pressure, peripheral vascular function and arterial stiffness between PRE‐ and E‐POST or L‐POST. Contrary to our hypothesis, cerebrovascular function was similar in post‐ compared with premenopausal females, with no detrimental vascular effects observed in the late postmenopausal group. However, this needs to be interpreted with caution because even non‐significant group differences in the peripheral vasculature have been shown to be associated with an increased risk of CVD events and mortality (Green et al., 2011). Furthermore, there were no associations between peripheral vascular or cerebrovascular function and estradiol or progesterone blood hormone concentrations. Blood hormones were also not associated with NO bioavailability or with vascular or cerebrovascular function.

Our findings are in contrast to previously reported differences in peripheral vascular and cerebrovascular function between pre‐, peri‐ and postmenopausal females (Brislane et al., 2020; Iwamoto et al., 2020; Moreau et al., 2012). Two previous studies describe a gradual reduction of brachial FMD and an increased arterial stiffness and blood pressure in post‐ compared with premenopausal females, respectively (Brislane et al., 2020; Moreau et al., 2012). We did not observe these differences in the peripheral vasculature in our sample of pre‐ and postmenopausal females. Our findings do align, in part, with the study by Brislane et al. (2020), who found no differences in brachial FMD in a sub‐analysis of late premenopausal (age, 46 ± 3 years) and early postmenopausal (age, 50 ± 3 years) females. However, when including the older postmenopausal group, brachial FMD was impaired in post‐ compared with premenopausal females (Brislane et al., 2020). Brachial FMD was >1% lower in the late postmenopausal group compared with the premenopausal group. Although this difference was not statistically significant, this difference in the percentage FMD might be clinical meaningful via increased CVD risk in the late postmenopausal group (Green et al., 2011). Additionally, we did not find any reduction in cerebrovascular function in the extra‐ or intracranial arteries at rest or in response to hypercapnia, whereas Iwamoto et al. (2020) reported lower reactivity of the ICA to hypercapnia in post‐ compared with premenopausal females. Furthermore, resting MCAv and CVCi, but not CVR, were also lower in post‐ compared with premenopausal females in the study by Brislane et al. (2020). However, when conducting a sub‐analysis with the late pre‐ and early postmenopausal females, they did not report any difference in MCAv or CVCi between groups (Brislane et al., 2020).

It is difficult to account for the isolated effects of menopausal status on vascular function owing to differences in the age groups between pre‐ and postmenopausal females. This was also observed in our findings, where the difference in ICA blood flow velocity change was no longer apparent after including age as a covariate. We aimed specifically to recruit participants from a relatively small age range (40–65 years) and included age as a covariate, in order to gain a better understanding of the impact of menopause, while minimizing the effect of age, and to address the gap in cerebrovascular research in females around the early stages of menopause (Alwatban et al., 2021; Ruediger et al., 2021). However, when factoring in estradiol as a covariate, the difference in ICA blood flow was no longer apparent either. Thus, it is not clear whether age or menopause or an interaction of both might cause changes in the cerebrovasculature.

Owing to the vasoactive capacities of oestrogen, it is suggested that declines in reproductive hormone levels during the menopausal transition and postmenopause might have a negative effect on peripheral vascular function. Our data showed no obvious effect of lower reproductive hormone levels on vascular function in healthy pre‐ and postmenopausal females. We also observed no association between reproductive hormone levels and NO bioavailability. This contrasts with Moreau et al. (2012), who assessed peripheral vascular function across the menopausal transition. They found that changes in peripheral vascular function begin during the menopausal transition and decline progressively throughout each stage of menopause (Moreau et al., 2012). Also, lower levels of estradiol and progesterone were associated with lower brachial FMD (Moreau et al., 2012). To isolate the effect of estradiol on vascular function, Moreau et al. (2020) reported declines in FMD in pre‐ and perimenopausal females after ovarian hormone suppression. The FMD returned to baseline levels in premenopausal females and increased to above baseline levels in the peri‐ and postmenopausal females when estradiol was restored after ovarian hormone suppression. These results support the concept that the decline in endothelial function across the menopausal transition is related to the reduction in estradiol. We did not report any significant associations between brachial FMD and estradiol in the present study. However, the influence and the different concentrations of other reproductive hormones (FSH and progesterone) in our groups make it challenging to investigate the isolated influence of estradiol on vascular and cerebrovascular function in the present study, especially given that FSH is strongly associated with central artery function (Waddell et al., 2001). In the study by Waddell et al. (2001), progesterone and oestrogen showed only weak correlations with arterial function. However, there was no strong correlation between peripheral artery function and any of the reproductive hormones (FSH, estradiol or progesterone). These results highlight that not only the decrease in oestrogen, but also the increase in FSH with female ageing might play a central role in arterial ageing in postmenopausal females (Waddell et al., 2001). A recent analysis of longitudinal data suggests that age rather than reproductive status influences cardiovascular risk factors, which appears in line with our findings (Raguindin et al., 2022). In the cerebral vasculature, a greater decline in MCAv across the lifespan has recently been reported in females compared with males, with the speed of decline being highest in females >60 years of age (Alwatban et al., 2021). This observation, along with our data, suggests that vascular changes might be preserved in younger postmenopausal females compared with older females.

Recent cross‐sectional data by Iwamoto et al. (2020) showed a lower ICA CVR from pre‐ to postmenopause. We did not see differences in ICA diameter or a change in shear rate in response to hypercapnia between groups in our sample, and, after accounting for age, there was no difference in ICA blood flow (in centimetres per second). The reported >44 year age gap between pre‐ and postmenopausal groups in the study by Iwamoto et al. (2020) compared with our 13 year age gap is likely to explain some the differences in our findings. However, Iwamoto et al. (2020) also reported an association between lower ICA reactivity and lower estradiol levels in pre‐, peri‐ and postmenopausal females, which was still present after adjustment for age. Age and estradiol levels were associated in our study, but we did not see any associations with estradiol and ICA CVR. However, when assessing logarithmically transformed data in only the postmenopausal group, estradiol and ICA CVR were positively associated. Physical activity might have influenced the findings in the present study, with all but three participants (one in each group) exceeding the current Australian physical activity guidelines. Given that exercise can also activate oestrogen‐related receptors, which, in turn, enable the release of vasoactive substances (Gliemann & Hellsten, 2019; Gliemann et al., 2019), this might explain, in part, why we did not see any strong association between estradiol and vascular function. Indeed, female vascular ageing is a multifactorial process and cannot be explained solely by the reduction in oestrogen. The involvement of other reproductive hormones (e.g., FSH and progesterone) makes it challenging to investigate the isolated influence of estradiol on vascular and cerebrovascular function. More longitudinal research is needed to assess the underlying mechanisms explaining why a loss of reproductive hormones may or may not cause changes in vascular function across the stages of menopause, including the potential protective effect of physical activity and exercise on vascular health.

### 4.1 Physical activity

The cohorts of pre‐ and postmenopausal females were more physically active compared with previous studies assessing differences in vascular outcomes. Previous studies reporting reduced vascular and cerebrovascular function in post‐ compared with premenopausal females also reported lower physical activity and/or lower aerobic fitness in the postmenopausal group (Brislane et al., 2020; Moreau et al., 2012) or excluded participants who were physically active (Iwamoto et al., 2020). In fact, we observed that the late postmenopausal group had the highest levels of self‐reported physical activity per week compared with our early postmenopausal and premenopausal groups (see Table 1). Although aerobic fitness was not specifically assessed in the present study, higher levels of physical activity are associated with a higher level of aerobic fitness (Bailey et al., 2013; Smith et al., 2021). In the peripheral vasculature, lower levels of aerobic fitness are associated with reduced vascular function across the stages of menopause (Moreau et al., 2012), and life‐long physical acitivity positively influences vascular function and the ability to form NO in postmenopausal females (Gliemann et al., 2019). Recently, Debray et al. (2022) reported no differences in PWV, brachial FMD and 24 h blood pressure, comparing physically active pre‐ and postmenopausal females, which is in line with our findings. Furthermore, a recent review and meta‐analysis highlights that aerobic exercise training improves FMD in postmenopausal females (Brislane et al., 2022). Likewise, elevated cardiorespiratory fitness is associated with increased MCAv and CVR when comparing trained and untrained individuals across different ages (Barnes et al., 2013; Smith et al., 2021). There are few studies assessing aerobic fitness and cerebral blood flow in ageing females, but a positive association between cardiorespiratory fitness and resting cerebrovascular conductance has been reported in postmenopausal females (age, 50–90 years) in one study (Brown et al., 2010). Furthermore, a recent study has highlighted that higher cardiorespiratory fitness was associated with a lower cerebral pulsatility index in females and males (age, 18–83 years), which remained positive in females only after adjusting for age (Zeller et al., 2022). There are conflicting results on whether exercise intervention studies might positively influence vascular function in postmenopausal females. The conflicting results might be attributable to many studies not accounting for age since the FMP or following the STRAW+10 criteria in categorizing menopausal status (Harlow et al., 2012). Thus, there might be variations in the responsiveness of the vasculature attributable to natural ageing and the progression of menopause. Maintaining a higher level of physical activity throughout menopause might preserve peripheral and cerebrovascular function in ageing females. Future research should include longitudinal studies assessing the effect of continuous physical activity throughout female ageing.

### 4.2 Limitations

The cross‐sectional nature of this study does not imply a causative link between the variables of interest. Despite no statistical difference in PWV or FMD, the magnitude of difference between groups is large and might have significance for clinical risks. When performing an a priori power calculation, we used ICA CVR as the main outcome and obtained an estimated *n* of 30 participants overall, with a modest effect size of 0.6 and a β‐power level of 0.8 as a result. More recent research (Brislane et al., 2020; Debray et al., 2022; Iwamoto et al., 2020), published after initializing the study, might have helped us to perform some more accurate power calculations for peripheral and cerebral outcomes. To explore the effects of oestrogen, ageing, physical activity and aerobic fitness, adequately powered longitudinal studies are needed. Given that hormone levels are inherently variable between females and between cycles within one person, measuring reproductive hormones is an unreliable method for categorizing menstrual cycle phase. As such, we had to rely on menstrual cycle tracking for the premenopausal group, which might have caused some inaccuracies. Physical activity was assessed using only the Active Australia Survey (AIHW, 2003), which increases the risk of over‐ or underestimation of physical activity by self‐report questionnaires. To improve the accuracy of the physical activity data, the survey was administered with a research staff member who was able to clarify any questions. However, using accelerometry or cardiopulmonary exercise testing might have allowed for a better understanding of the impact of physical activity and aerobic fitness on the findings.

## 5 CONCLUSION

We observed no differences in vascular function between healthy, active pre‐ and postmenopausal females. After accounting for age, there was no difference in cerebrovascular function between groups, and there were no associations between reproductive hormone levels, vascular function and NO bioavailability. More research is needed in this area to monitor the effect of a gradual decline in oestrogen throughout menopause on vascular and cerebrovascular function and to investigate the effects of physical activity and aerobic fitness on maintenance of peripheral and cerebrovascular health throughout the menopausal transition.

## AUTHOR CONTRIBUTIONS

Stefanie L. Ruediger, Jeff S. Coombes and Tom G. Bailey conceived the study design. Stefanie L. Ruediger, Jodie L. Koep and Faith K. Pizzey were involved with data collection. Stefanie L. Ruediger analysed all data, which was checked for accuracy by Faith K. Pizzey and Jodie L. Koep. Stefanie L. Ruediger drafted the work. Christopher D. Askew, Tom G. Bailey and Jeff S. Coombes contributed to critical revisions of the early draft. All authors contributed to critical review and revisions of the manuscript. All authors approved the final manuscript and agree to be accountable for all aspects of the work in ensuring that questions related to the accuracy or integrity of any part of the work are appropriately investigated and resolved. All persons designated as authors qualify for authorship, and all those who qualify for authorship are listed.

## CONFLICT OF INTEREST

None declared.

## Supporting information



Statistical Summary Document

Supporting Information

## Data Availability

All data supporting the results of this paper are reported in the manuscript (Tables 1, 2, 3, 4; Figure 1). The entire raw data sets that support the findings of this study are available on request from the corresponding author. The data are not publicly available owing to ethical restrictions.
