# Supplementary material for: Comparison of peripheral and cerebral vascular function between premenopausal, early and late postmenopausal females
Source: Exp Physiol. 2023 Jan 9;108(3):518–30. doi: 10.1113/EP090813 (PMC10103882; doi:10.1113/EP090813)
Supplement: Supplementary file 2 — Supporting Information [file EPH-108-518-s001.docx]

Supplementary material

Figure 2a: Scatterplot of brachial flow mediated dilation (FMD) and estradiol (E2)

Figure 2b: Scatterplot of brachial flow mediated dilation (FMD) and progesterone (PG)

Figure 2c: Scatterplot of brachial flow mediated dilation (FMD) and follicle stimulating hormone (FSH)

Figure 3a: Scatterplot of cerebrovascular reactivity of the ICA (ICA CVR) and estradiol (E2)

Figure 3b: Scatterplot of cerebrovascular reactivity of the ICA (ICA CVR) and progesterone (PG)

Figure 3c: Scatterplot of cerebrovascular reactivity of the ICA (ICA CVR) and follicle stimulating hormone (FSH)
